# SEOM-GOTEL clinical guidelines on diffuse large B cell lymphoma (2022)

**DOI:** 10.1007/s12094-023-03206-5

**Published:** 2023-06-08

**Authors:** Josep Gumà, Natalia Palazón-Carrión, Antonio Rueda-Domínguez, Silvia Sequero, Virginia Calvo, Ramón García-Arroyo, José Gómez-Codina, Marta Llanos, Natividad Martínez-Banaclocha, Mariano Provencio

**Affiliations:** 1 Medical Oncology Department, Hospital Universitari Sant Joan de Reus, IISPV, URV, Reus, Tarragona, Spain; 2 Medical Oncology Department, Hospital Universitario Virgen de la Macarena, Seville, Spain; 3 UGCI Medical Oncology, Hospitales Universitarios Regional y Virgen de la Victoria, IBIMA, Málaga, Spain; 4 Medical Oncology Department, Hospital Universitario San Cecilio, Granada, Spain; 5 Medical Oncology Department, Hospital Universitario Puerta de Hierro, Majadahonda, Madrid, Spain; 6 Medical Oncology Department, Complejo Hospitalario Universitario, Pontevedra, Spain; 7 Medical Oncology Department, Hospital Universitari i Politècnic La Fe, Valencia, Spain; 8 Medical Oncology Department, Hospital Universitario de Canarias, Tenerife, Spain; 9 Oncology Department, Hospital General Universitario Dr. Balmis, Institute for Health and Biomedical Research (ISABIAL), Alicante, Spain

**Keywords:** Diffuse large B-cell lymphoma, Guideline, Diagnosis, Treatment

## Abstract

Diffuse large B-cell lymphoma is the most frequent histological subtype of NHL and the paradigm for the management of aggressive lymphoma. An excisional or incisional lymph node biopsy evaluated by an experienced hemopathologist is recommended to establish the diagnosis. Twenty years following its introduction, R-CHOP remains the standard first-line treatment. No modification of this scheme (increased chemotherapy dose intensity, new monoclonal antibodies, or the addition of immunomodulators or anti-target agents) has significatively improved the clinical outcomes, whereas therapy for recurrence or progression is evolving rapidly. The irruption of CART cells, polatuzumab vedotin, tafasitamab, and CD20/CD3 bispecific antibodies are changing the natural history of relapsed patients and will challenge R-CHOP as the benchmark for newly diagnosed patients.

## Methodology

This guideline is based on relevant published studies and with the consensus of ten GOTEL (Oncology Group for the Treatment and Study of Lymphomas) and SEOM (Spanish Society of Medical Oncology) lymphoma treatment expert oncologists. The Infectious Diseases Society of America-US Public Health Service Grading System for Ranking Recommendations in Clinical Guidelines [1] has been used to assign levels of evidence and grades of recommendation.

### Incidence and epidemiology

In Spain, non-Hodgkin’s lymphoma (NHL) is the seventh most common tumor in men and eighth in women [2]. As in other Westernized countries, its incidence increased by 6% per year between 1975 and 1995 for unknown reasons and, since then, has remained stable until today [3].

In our setting, DLBCL is the most widespread histological subtype of NHL (30%), followed by follicular lymphoma and marginal cell lymphoma, and is the paradigm of aggressive lymphoma. The estimated number of cases of DLBCL diagnosed in Spain in 2021 was 6,933, with a crude incidence rate of 5.53 cases/100,000 inhabitants/year. The median age at diagnosis was 68 years and, as with most hematological neoplasms, it was more frequent in men (sex ratio 1.37) [4, 5]. During the 2002–2013 period, the five-year relative survival of DLBCL in Spain was 55.6% (REDECAN; unpublished data).

### Diagnosis, pathology, and molecular biology

The diagnosis of lymphoma is based on a comprehensive evaluation of histologic, immunophenotypic, cytogenetic, and molecular studies that are interpreted within the context of the clinical backdrop. The differential diagnosis of DLBCL includes other entities that can result in lymphadenopathy and similar pathologic features. Patients usually present a growing mass in any adenopathic territory or symptoms of infiltration of extralymphatic organs. One-third have systemic “B” symptoms (i.e., fever, weight loss, sweats), and 40% have extranodal disease.

When available, a lymph node is generally the preferred tissue for diagnosis. Biopsy specimens should include both fresh and formalin-fixed material and should be evaluated by an experienced hematopathologist. If nodal involvement is not identified, diagnosis can be made using extranodal tissue. The tissue sample should be obtained urgently if aggressive NHL is suspected and, ideally, prior to steroid treatment.

An excisional or incisional lymph node biopsy is recommended to establish the diagnosis of DLBCL. A core needle biopsy is not optimal, but can be used in certain clinical circumstances. Importantly, fine-needle aspiration alone is not an acceptable substitute for an initial diagnosis of lymphoma, although it may suffice to diagnose recurrence [6].

In addition to morphological classification, pathology assessment must necessarily include immunohistochemistry (IHC), flow cytometry, fluorescent in situ hybridization (FISH), and molecular tests. Three main morphologic subtypes of DLBCL (centroblastic, immunoblastic, and anaplastic) have been recognized, albeit with poor reproducibility among pathologists and scant clinical relevance. IHC core panel for DLBCL diagnosis includes CD20, CD3, CD5, CD10, CD45, BCL2, BCL6, Ki-67, IRF4/MUM1, and MYC. CD30, HHV-8, and EBV may be needed for classification.

Gene expression profiling (GEP) classifies DLBCL into three biological subtypes that arise from the developmental stages of normal B cells or their so-called ‘cell of origin’ (COO): germinal center B-cell-like (GCB) (56%), activated B-cell-like (ABC) (32%), and type 3 (11%). Retrospective studies have revealed significantly better outcomes for GCB vs ABC. That being said, prospective studies have failed to detect significant differences in terms of survival. Furthermore, COO alone does not explain the clinical heterogeneity across patients and up to 20% of DLBCL cannot be classified using this genetic signature [7].

Hans’ immunohistochemical algorithm based on CD10, BCL6, and IRF4/MUM1 differentiates COO more quickly and cost-effectively, but does not identify the entire ABC subgroup and there is insufficient scientific evidence to be of use for treatment decisions [8].

Genomic sequencing platforms classify DLBCL into groups with different genetic signatures that are related to treatment outcomes. Nevertheless, these genetic models also leave a proportion of cases unclassified and are not currently available in routine clinical practice [9].

FISH analysis of aggressive B-cell lymphomas is needed to detect MYC and BCL2/BCL6 translocations, as they define a new entity in the latest WHO classification (“High-grade B-cell lymphoma with MYC and BCL2 and/or BCL6 rearrangements”), which more often than not belong to the GCB. These patients have particularly poor outcomes with R-CHOP, making them good candidates for inclusion in clinical trials. DLBCL overexpressing MYC and BCL-2 (double expressor) tend to be included in the ABC group and should not be confused with double-hit or triple-hit (DH/TH) high grade lymphomas that carry genetic translocations [10].

#### Recommendations

- Excisional/incisional biopsy of the adenopathy or affected extranodal tissue is the method of choice for the diagnosis of DLBCL. **[IA]**
- The combination of clinical data with histopathological, immunohistochemical, and cytogenetic/molecular study enable DLBCL to be categorized according to the standard WHO system. The basic panel for IHC in DLBCL includes CD20, CD3, CD5, CD10, CD45, BCL2, BCL6, Ki-67, IRF4/MUM1, and MYC. **[IA]**
- An immunophenotyping algorithm is required to establish the COO (Hans, etc.), albeit, outside of a clinical trial, there is currently insufficient clinical evidence upon which to base a therapeutic decision. GEP studies are not deemed standard when diagnosing DLBCL in clinical practice. **[IIB]**
- Tissue that is IHC positive for MYC, BCL2, or BCL6 expression should undergo FISH to detect MYC, BCL2, and BCL6 gene rearrangements. **[IIA]**
- In the absence of prospective studies displaying improved outcomes based on targeted treatment, NGS cannot be recommended in routine clinical practice. **[IVC]**

### Staging and risk assessment

The staging process should include a complete medical history, physical examination, laboratory tests, and imaging studies according to the *Recommendations for Initial Evaluation, Staging, and Response Assessment of Hodgkin and Non-Hodgkin Lymphoma* of The Lugano Classification [11]:

- Anamnesis with special attention to the presence of B symptoms (fever, profuse night sweats, and loss of more than 10% of body weight the last 6 months) and signs suggestive of extralymphatic involvement, in addition to ECOG performance status.
- Complete physical examination, especially of all peripheral lymph node regions, Waldeyer’s ring, and skin.
- Routine laboratory studies including complete blood count, electrolytes, kidney and liver function tests, those necessary to determine prognostic indices (serum LDH and beta-2-microglobulin), as well as serologies for hepatitis B and C, and HIV.
- PET-CT with iodinated contrast is the imaging test *par excellence* to stage aggressive lymphomas and will serve as a baseline reference for treatment response.
- Because PET-CT can detect bone marrow involvement in DLBCL, bone marrow biopsy is not routinely recommended [12].
- Cerebrospinal fluid cytology for subjects at risk for CNS involvement (High-risk IPI, kidney or adrenal involvement, or primary testicular and breast DLBCL).
- Echocardiogram or radionuclide ventriculogram in individuals at risk for heart failure if anthracyclines or mediastinal radiotherapy are indicated.
- Inform the patient about possible fertility preservation techniques before the start of chemotherapy

For staging, the Lugano modification of the Ann Arbor classification system is used (Table 1). Note that the suffixes A and B are no longer applied in this staging system. There is no consensus regarding the cut-off point to define bulky disease, ranging from 7.5 to 10 cm.

**Table 1:** Revised staging system for primary nodal lymphomas (Lugano classification)

Stage	Involvement	Extranodal status
I	One node or a group of adjacent nodes	Single extranodal lesions without nodal involvement
II	Two or more nodal groups on the same side of the diaphragm	Stage I or II by nodal extent with limited contiguous extranodal involvement
II bulky*	II as above with “bulky” disease	Not applicable
III	Nodes on both sides of the diaphragm; nodes above the diaphragm with spleen involvement	Not applicable
IV	Additional noncontiguous extralymphatic involvement	Not applicable

Extent of disease is determined by positron emission tomography–computed tomography. Tonsils, Waldeyer’s ring, and spleen are considered nodal tissue. *Whether stage II bulky disease is treated as limited or advanced disease may be determined by histology and a number of prognostic factors

The International Prognostic Index (IPI), with five variables (including stage), improves prognostic accuracy over stage alone (Table 2). Despite the original International Prognostic Index [13] being conceived in the pre-rituximab era, more recent studies have validated this score for patients treated with chemotherapy plus rituximab. For those treated with CHOP or CHOP-like chemotherapy plus rituximab, three-year OS (overall survival) for Low, Low-intermediate, High-intermediate, and High-risk groups was 91, 81, 65, and 59 percent, respectively [14].

**Table 2:** International prognostic index

Adverse prognostic factors	Risk group
Age > 60	
Serum LDH > normal	Low-risk: 0–1
ECOG ≥ 2	Low-intermediate: 2
Stage III-IV	High-intermediate: 3
Extranodal involvement > 1 site	High: 4–5

People with limited states of DLBCL have a markedly better prognosis and the IPI has been modified for this subgroup (see below) [15, 16].

#### Recommendations

- The initial evaluation and staging should be performed according to the Lugano Classification, which establishes PET-CT as the recommended imaging test for staging and response assessment **[IA].**
- Bone marrow biopsy is not routinely recommended **[IIA]**.
- The original International Prognostic Index (IPI) should be used to appraise the risk of treatment failure **[IA].**

### Treatment of early-stage

Limited-stage DLBCL is commonly defined as Ann Arbor stages I and II, usually anatomically located in a potential radiation field. Standard treatment consists of chemoimmunotherapy with R-CHOP (rituximab, cyclophosphamide, doxorubicin, vincristine, and prednisone) alone or in combination with involved-site radiation therapy (ISRT).

Radiotherapy alone is not an acceptable treatment for limited-stage DLBCL, with the exception of a selected group of patients with stage I and comorbidities in whom the use of chemotherapy may be contraindicated.

To select the most appropriate treatment, subjects with limited-stage DLBCL can be stratified based on the presence of adverse prognostic factors according the so-called mIPI or stage-modified version of IPI [16] (the same prognostic factors reported by Shipp et al., except that the adverse risk factor for limited stage is stage II, as opposed to stages III-IV):

- Stage II
- Serum LDH above normal limits
- Age > 60
- ECOG ≥ 2

Moreover, the presence or absence of bulky disease (defined as a tumor > 7.5 or 10 cm) must be taken into account.

Stages I without any adverse prognostic factor have a 5-year median survival > 90%. Stages I and II with some adverse prognostic factor have a 5-year OS of 70%. However, in bulky disease, OS decreases to 50% at 5 years (similar to advanced stages).

For practical purposes, early stages of DLBCL can be divided into three groups:

- Group I: 0–1 adverse prognostic factors, non-bulky.
- Group II: ≥ 2 adverse prognostic factors, non-bulky.
- Group III: bulky disease

For group I patients, treatment with four cycles of R-CHOP is usually enough. There are two trials that endorse this strategy. The FLYER phase 3, non-inferiority trial included 592 patients ≤ 60 years with stage I-II DLBCL without adverse prognostic factors. Four cycles of R-CHOP followed by two doses of rituximab alone were compared to six cycles of R-CHOP. No differences were found in terms of 5-year OS, progression-free survival (PFS), and event-free survival (EFS); toxicity was lower with four cycles of R-CHOP [17]. In the LYSA trial, 334 patients without bulky disease received four cycles of R-CHOP-14 if mIPI = 0 and six cycles if mIPI ≥ 1, with or without 40 Gy ISRT. Again, no differences were detected in EFS and OS; similarly, no benefit was derived from the addition of radiotherapy [18].

In group II, two options might be acceptable, depending on whether a risk-adapted therapeutic approach is adopted, that is, based on an interval PET.

If a risk-adapted approach is not considered, three cycles of R-CHOP followed by radiation therapy (30 Gy ISRT) or six cycles of R-CHOP are both suitable treatment alternatives [15, 19].

Although no head-to-head studies of risk-adapted therapy *versus* conventional treatment in any stage of DLBCL [20], some authors propose the following recommendations:

- If PET-negative after three cycles of R-CHOP, complete treatment with one additional cycle of the same chemotherapy.
- If PET-positive after three cycles of R-CHOP, complete treatment with either three additional cycles of the same chemotherapy or 36–40 Gy of ISR [21].

Prioritization of more cycles of chemotherapy over radiotherapy or vice versa should be based on the individual’s comorbidities and probability of long-term toxicities.

The therapeutic approach for cases of bulky disease (group 3) should be the same as in advanced disease although, in this scenario, radiotherapy may play a more relevant role. Administration of six cycles of R-CHOP followed by 30–40 Gy ISRT is generally recommended [22, 23].

#### Recommendations

- 0–1 mIPI adverse prognostic factors: R-CHOP × 4 **[IIA]**
-  ≥ 2 mIPI adverse prognostic factors:
  - Conventional treatment: R-CHOP × 3 + RT or R-CHOP × 6 **[IIA]**
  - Risk-adapted therapy **[IIC]**
    - R-CHOP × 4 if PET3-negative
    - R-CHOP × 6 or R-CHOP × 3 + RT if PET3-positive
- Bulky disease (≥ 7.5–10 cm):
  - R-CHOP × 6 + RT **[2A]**.

### Treatment of advanced-stage

Approximately 70% of patients have advanced-stage disease at diagnosis. The standard of care in first-line therapy of advanced-stage diffuse large B-cell lymphoma (DLBCL) remains R-CHOP every 21 days (R-CHOP-21). This is based on the results of the phase III study by the GELA group, in which 8 cycles of R-CHOP in patients > 60 years of age significantly improved survival compared to 8 cycles of CHOP. This survival benefit has been upheld in subsequent trial updates: 5-year OS for patients treated with CHOP and R-CHOP was 45% and 58%, respectively (*p* = 0.0073) [24]. In the MiNT trial, individuals < 60 years were randomized to receive either 6 cycles of CHOP-like or 6 cycles of R-CHOP-like treatment; six-year EFS was 55.8% vs. 74.3% (*p* < 0.0001) [22].

Increasing drug density, in the RICOVER-60 trial, R-CHOP administered every 14 days (R-CHOP-14) significantly improved survival over CHOP-14 [25]. Subsequently, the two large randomized trials comparing R-CHOP-21 with R-CHOP-14 revealed no survival benefit [26, 27].

With the aim of improving R-CHOP outcomes, several studies have been conducted increasing chemotherapy dose intensity. A randomized study by the French group, in subjects aged 18–59 with an age-adjusted IPI score (aaIPI) 1, a dose-intensive regimen of rituximab combined with doxorubicin, cyclophosphamide, vindesine, bleomycin, and prednisone (R-ACVBP) with subsequent consolidation using methotrexate, rituximab-ifosfamide-etoposide, and cytarabine has been the only regimen providing a survival advantage over R-CHOP. The 3-year OS rate was 92% vs. 84%, albeit at the expense of much greater toxicity [28]. In a phase II study, high-risk cases (IPI 3–5, aaIPI 2–3) were treated with DA-EPOCH-R (adjusted dose of etoposide-prednisone-vincristine-cyclophosphamide-doxorubicin-rituximab), achieving DFS and OS at 10 years of 47.8% and 63.6%, respectively [29]. However, in a randomized study (ALLIANCE/CALGB50303 trial) comparing R-CHOP with DA-EPOCH-R, the latter was associated with greater toxic effects and no benefit in PFS or OS [30].

Another strategy in the first-line treatment of advanced-stage DLBCL was to change the type of monoclonal antibody associated to CHOP. The GOYA randomized, phase III study compared R-CHOP with obinutuzumab-CHOP and detected no PFS differences [31]. Polatuzumab is an anti-CD79b antibody conjugated to monomethyl auristatin, an anti-tubulin agent. In the POLARIX randomized, phase III, clinical trial, 879 treatment-naïve patients with intermediate-risk or high-risk DLBCL were randomized to receive 6 cycles of either R-CHOP or polatuzumab-R-CHP [32]. Two-year PFS was 76.7% in the experimental arm and 70.2% in the control arm, HR 0.73 (95% CI, 0.57–0.95); *p* = 0.02. OS was similar in both arms, although more patients in the control arm required salvage treatment. Toxicity was similar, and the potential higher benefit for the activated B-cell-like subtype is suggested in the subgroup analysis with a HR of 0,4, although these results must be taken cautiously as patients were not stratified by this characteristic..

Various randomized trials have evaluated the addition of new agents to R-CHOP: bortezomib, ibrutinib, venetoclax, or lenalidomide, however, without yielding any statistical differences. Likewise, several phase III trials have failed to demonstrate a survival benefit of post-R-CHOP maintenance therapy, with agents such as rituximab, enzastaurin, everolimus, or lenalidomide.

#### Recommendations

- Six cycles of R-CHOP-21 remains the standard of care for advanced-stage DLBCL. Its replacement by Polatuzumab-CHP is a possibility to take into account in the future, if this combination is approved by the Spanish regulatory agencies. **[IA]**
- The value of consolidative radiation therapy after immunochemotherapy has not been proved. **[IVC]**

**Figure:**
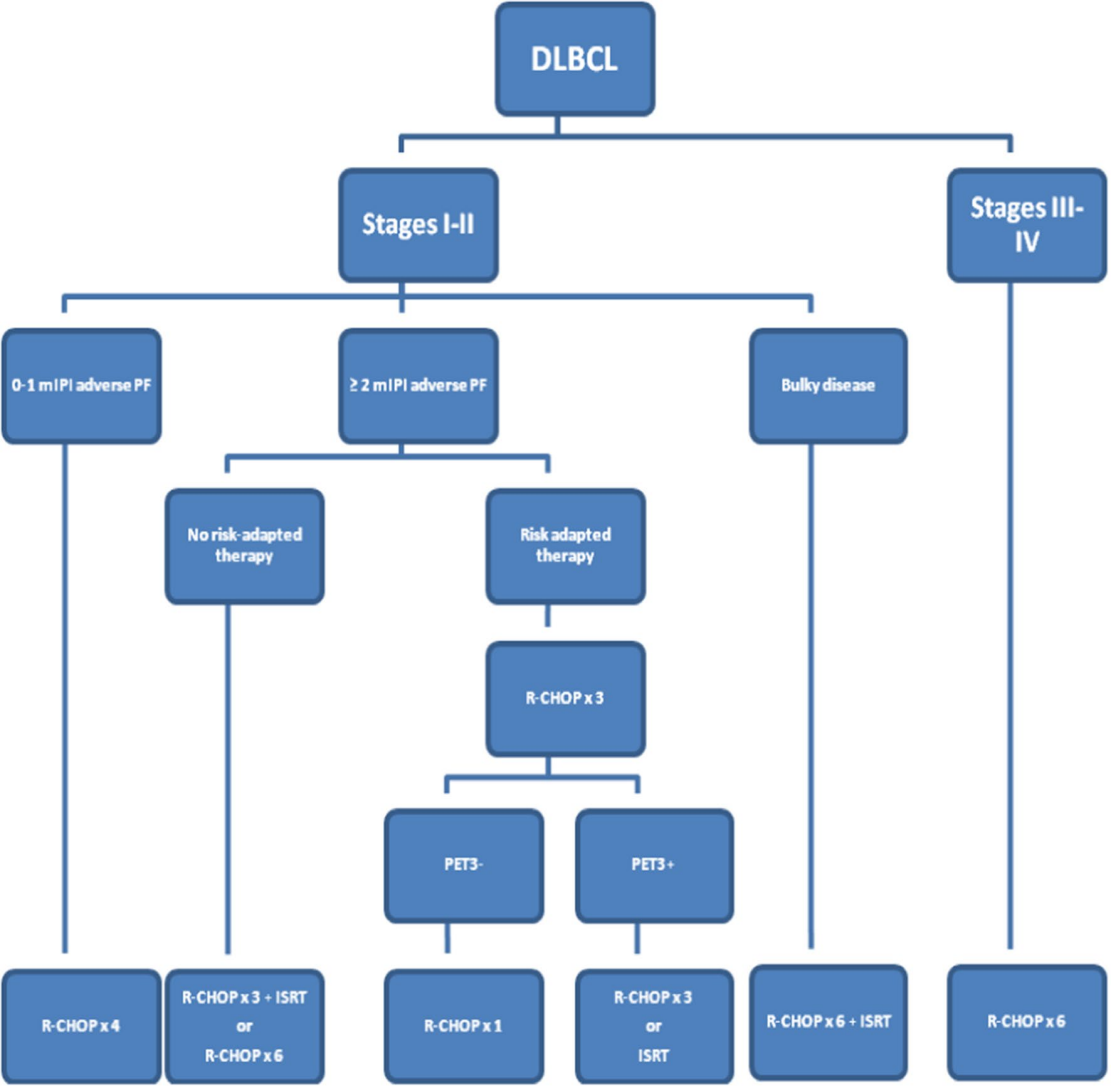
**Treatment algorithm for newly diagnosed DLBCL** DLBCL: diffuse large B-cell lymphoma; mIPI: stage-modified International Prognostic Index; PF: prognostic factors; R-CHOP: rituximab, cyclophosphamide, doxorubicin, vincristine, prednisone (repeat cycles at 21-day intervals); PET3: PET after the third cycle of CT; ISRT: involved-site radiotherapy.

### Treatment of refractory-relapsed disease

Unfortunately, 10–15% of patients do not respond to initial treatment (primary refractory disease) and 20–30% will relapse after achieving a complete response.

Salvage regimens are recommended with rituximab and chemotherapy (R-DHAP, R-ICE, R-GDP, R-ESHAP) to be followed by high-dose chemotherapy (HDC) and autologous hematopoietic cell transplantation (HCT) in responsive patients. R-DHAP (rituximab, cisplatin, cytarabine, dexamethasone) or R-ICE (rituximab, ifosfamide, carboplatin, etoposide) appear to yield similar outcomes as salvage regimens [33]. However, in the LY.12 trial, R-GDP (rituximab, cisplatin, gemcitabine, dexamethasone) demonstrated similar efficacy, but less toxicity than R-DHAP [34]. For selected cases with documented CNS involvement, high-dose cytarabine-based salvage regimens (R-ESHAP, R-DHAP) can be considered, but no studies have directly compared high-dose cytarabine-based salvage therapy with other intensive regimens for r/r DLBCL. BEAM (carmustine, etoposide, cytarabine, and melphalan) is the most commonly used high-dose regimen in the absence of randomized studies that demonstrate the superiority of either. Response should be evaluated by FDG-PET scan after three to four cycles of the salvage regimen (before HDC) and after completion of all therapy.

Results have been dissimilar when CAR-T therapy is used as second-line treatment in r/r DLBCL, compared to standard treatment with salvage chemotherapy followed by HDC. In three published phase III studies in primary refractory or DLBCL progressing within 12 months of first-line therapy [35–37], axicabtagene ciloleucel and lisocabtagene maraleucel demonstrated better results than HDC, while these differences did not materialize when HDC and tisagenlecleucel were compared. These discordant results may be due to differences in time to CAR-T infusion (29 days with axi vs 52 days with tisa) and the possibility of using bridging therapy in the trial with tisagenlecleucel (this may have conditioned the inclusion of patients with clinically more aggressive disease and worse prognosis). Thus, it appears that second-line CART therapy might be restricted to patients with early progression and no immediate need for treatment. Therefore, second-line CAR-T therapy cannot be recommended at this time, except for those subjects who do not respond to pre-transplant second-line chemotherapy.

Patients not suitable for high-dose therapy and autologous HCT may be treated with the aforementioned salvage regimens or others, such as R-GEMOX (rituximab, gemcitabine, oxaliplatin). Nevertheless, they should be preferably enrolled in clinical trials. Newer treatment options include anti-CD20 in combination with anti-CD79b mAbs (polatuzumab-rituximab-bendamustine) and combinations with anti-CD19 mAb (e.g., tafasitamab + lenalidomide), exhibiting promising activity even in cases of disease that was refractory to previous CD20-targeted immunochemotherapies [38, 39].

Individuals who relapse or fail to respond to second-line chemotherapy and autologous HCT are candidates for treatment with CAR-T cells if they maintain a good performance status. Third-line CAR-T therapy in r/r DLBCL achieves objective responses in 50–80%, with long-term survival around 40%. However, neurological toxicity and cytokine release syndrome are potentially life-threatening adverse effects that must be contemplated [40–42]. Axicabtagene ciloleucel (axi-cel) and tisagenlecleucel (tisa-cel) are the two commercially available, anti-CD19, CAR-T products for this indication in Spain.

Unfortunately, at least 50% of patients who relapse or are refractory to two or more lines of therapy are also refractory or relapse following CAR-T-cell therapy. In these cases, there is no optimal standard of care; hence, it is advisable that they be enrolled in clinical trials. Preliminary data from ongoing, early phase clinical trials with anti-CD20/CD3 bispecific mAb (mosunetuzumab, glofitamab, epcoritamab, and odronextamab) display promising efficacy and a favorable safety profile in subjects with heavily pretreated r/r DLBCL, including those who have received prior CAR-T-cell therapy. At present, and in view of the activity of new drugs (e.g., bispecific antibodies, polatuzumab vedotin, tafasitamab…) allogeneic transplant is only an experimental alternative for selected cases after multiple relapses. Individuals who are not candidates for combined treatment can be treated with monotherapy agents (pixantrone, rituximab, gemcitabine, oxaliplatin, etc.) with poor response rates (< 20%), albeit with some symptomatic benefit, or palliative radiotherapy.

#### Recommendations

- The first relapse or refractoriness of DLBCL in a fit patient should be treated with second-line chemotherapy followed by HDC, in those patients showing chemosensibility. **[IIA]**
- Subjects in first r/r DLBCL not suitable for high-dose chemotherapy should be treated with conventional second-line chemotherapy. **[IIIA]**
- Currently, the use of CAR-T therapy in first recurrence of DLBCL is not yet justified, although this recommendation may be modified in the near future. **[IIC]**
- Patients who do not respond or who relapse after HDC could be candidates for CAR-T therapy. **[IIIA]**
- Recurrences after HDC and CAR-T therapy or patients who are not candidates for CAR-T could be treated with bendamustine-rituximab-polatuzumab, tafasitamab-lenalidomide, pixantrone, or low-dose palliative conventional chemotherapy. Treatment with bispecific antibodies or other new drugs in a clinical trial regimen is highly recommended. **[IIIA]**

**Figure:**
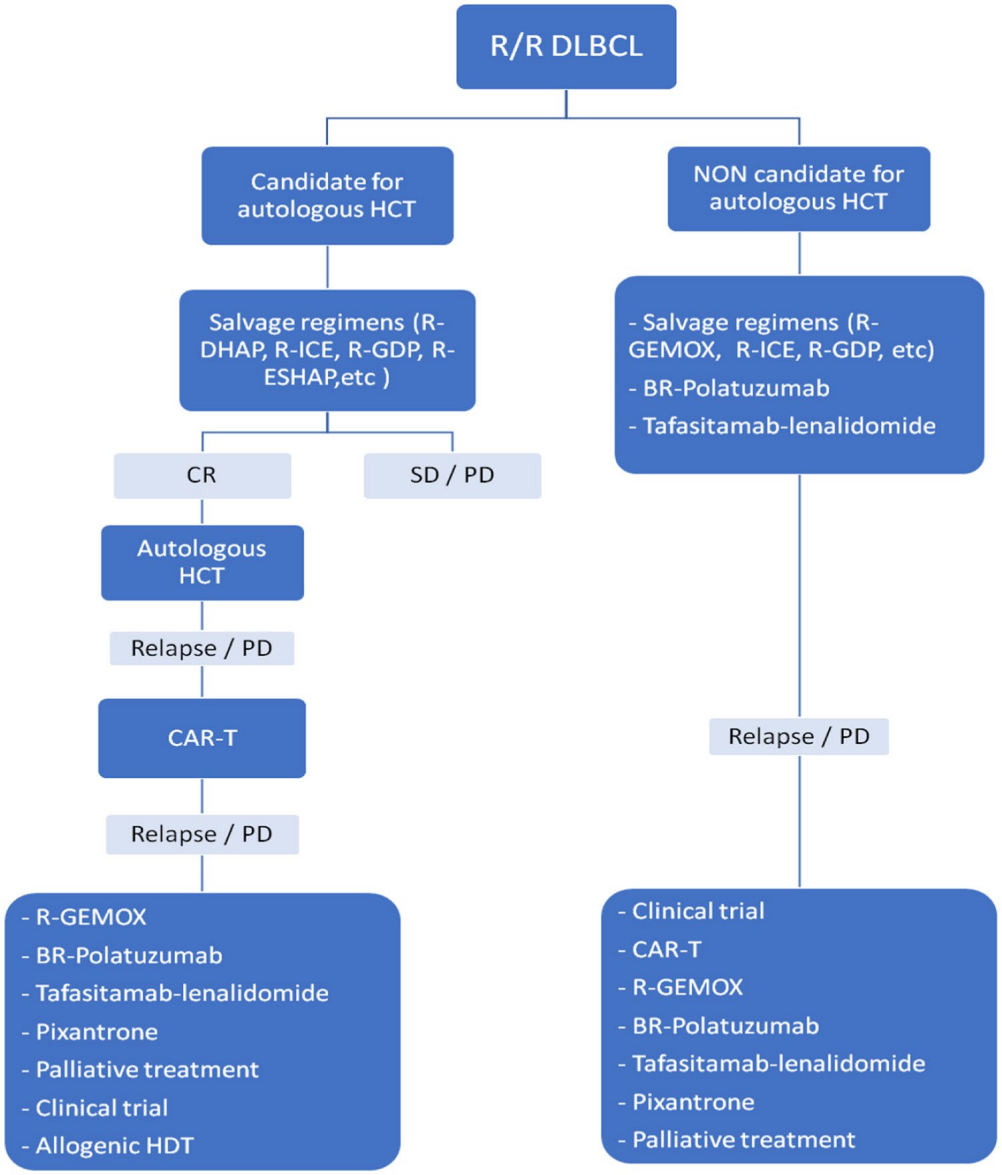
**Treatment algorithm for R/R DLBCL** R/R DLBCL: refractory-relapsed diffuse large B-cell lymphoma; CR: complete response; PR: partial response; SD: stable disease; PD: progressive disease; HCT: hematopoietic cell transplantation; BR: rituximab, bendamustine; CAR-T: chimeric antigen receptor T-cell; R-GEMOX: rituximab, gemcitabine, oxaliplatin; R-DHAP: rituximab, cisplatin, cytarabine, dexamethasone; R-ICE: rituximab, ifosfamide, carboplatin, etoposide; R-GDP: rituximab, cisplatin, gemcitabine, dexamethasone; R-ESHAP: etoposide, methyl-prednisolone, cytarabine, cisplatin.

## Follow-up, long-term implications, and survivorship

Patients who achieve a complete response to first-line treatment should undergo follow-up based primarily on history and clinical examination. Periodic blood tests are also recommended and may be helpful when recurrence is suspected and to detect possible late treatment toxicities. In asymptomatic cases, routine imaging techniques, such as CT, have not been shown to be useful in terms of survival [43, 44], although many oncological and hematological teams do it so for the first two or three years.

In women who have received mediastinal radiotherapy, especially before the age of 25, a breast MRI can be considered beginning 8–10 years after treatment. In any case, with the use of modern radiotherapy techniques and small fields (ISRT), the risk of radiation-induced breast cancer seems lower. Smoking cessation counseling is important for all patients, especially those who have received radiation therapy to the head and neck and chest.

### Recommendations

- Anamnesis and clinical examination every three months the first year, every 6 months the second and third years, and once a year thereafter. **[IVA]**
- Blood tests, including complete blood count, LDH, and ESR, every 6 months for the first two years and annually thereafter. **[IVA]**
- Routine imaging tests are not recommended in the absence of clinical suspicion of recurrence.** [IIB]**
- Consider yearly breast MRI in women treated with thoracic radiotherapy starting at 8 years post-treatment. **[IVB]**
- Advice about smoking cessation.** [IVA]**

## Data Availability

Not applicable : qualitative interview data will not be shared in order to protect the identity of the study participants.
